# Selection strategy of phage-displayed immunogens based on an in vitro evaluation of the Th1 response of PBMCs and their potential use as a vaccine against *Leishmania infantum* infection

**DOI:** 10.1186/s13071-017-2576-8

**Published:** 2017-12-21

**Authors:** Fernanda Fonseca Ramos, Lourena Emanuele Costa, Daniel Silva Dias, Thaís Teodoro Oliveira Santos, Marcella Rezende Rodrigues, Daniela Pagliara Lage, Beatriz Cristina Silveira Salles, Vívian Tamietti Martins, Patrícia Aparecida Fernandes Ribeiro, Miguel Angel Chávez-Fumagalli, Ana Carolina Silva Dias, Patrícia Terra Alves, Érica Leandro Marciano Vieira, Bruno Mendes Roatt, Daniel Menezes-Souza, Mariana Costa Duarte, Antonio Lúcio Teixeira, Luiz Ricardo Goulart, Eduardo Antonio Ferraz Coelho

**Affiliations:** 1 Programa de Pós-Graduação em Ciências da Saúde: Infectologia e Medicina Tropical, Faculdade de Medicina, Universidade Federal de Minas Gerais, Av. Prof. Alfredo Balena, 190, Belo Horizonte, Minas Gerais 30130-100 Brazil; 2 Laboratório de Nanobiotecnologia, Instituto de Genética e Bioquímica, Universidade Federal de Uberlândia, Av. Amazonas s/n, Campus Umuarama, Bloco 2E, Sala 248, Uberlândia, Minas Gerais 38400-902 Brazil; 3 Laboratório Interdisciplinar de Investigação Médica, Faculdade de Medicina, Universidade Federal de Minas Gerais, Av. Prof. Alfredo Balena, 190, Belo Horizonte, Minas Gerais 30130-100 Brazil; 4 Departamento de Patologia Clínica, COLTEC, Universidade Federal de Minas Gerais, Av. Antônio Carlos, 6627, Pampulha, Belo Horizonte, Minas Gerais 31270-901 Brazil; 5 Neuropsychiatry Program, Department of Psychiatry and Behavioral Sciences, McGovern Medical School, The University of Texas Health Science Center at Houston, 1941 East Road, Houston, TX 77041 USA; 6 Department of Medical Microbiology and Immunology, University of California-Davis, Davis, CA 95616 USA

**Keywords:** Phage display, Peripheral blood mononuclear cells, Antibodies, Immune response, Vaccine, Visceral leishmaniasis

## Abstract

**Background:**

The development of a vaccine for the prevention of visceral leishmaniasis (VL) still represents a significant unmet medical need. A human vaccine can be found if one takes into consideration that many people living in endemic areas of disease are infected but do not develop active VL, including those subjects with subclinical or asymptomatic infection.

**Methods:**

In this study, a phage display was used to select phage-exposed peptides that were specific to immunoglobulin G (IgG) antibodies from asymptomatic and symptomatic VL patients, separating them from non-infected subjects. Phage clones presenting valid peptide sequences were selected and used as stimuli of peripheral blood mononuclear cells (PBMCs) obtained from both patients’ groups and controls. Those with higher interferon-gamma (IFN-γ)/interleukin (IL)-10 ratios were further selected for vaccination tests.

**Results:**

Among 17 evaluated clones, two were selected, B1 and D11, and used to immunize BALB/c mice in an attempt to further validate their in vivo protective efficacy against *Leishmania infantum* infection. Both clones induced partial protection against the parasite challenge, which was evidenced by the reduction of parasitism in the evaluated organs, a process mediated by a specific T helper (Th)1 immune response.

**Conclusions:**

To the best of our knowledge, this study is the first to use a rational strategy based on in vitro stimulation of human PBMCs with selected phage-displayed clones to obtain new immunogens against VL.

**Electronic supplementary material:**

The online version of this article (10.1186/s13071-017-2576-8) contains supplementary material, which is available to authorized users.

## Background

Leishmaniases are diseases caused by the infection with *Leishmania* protozoan parasites, visceral leishmaniasis (VL) representing the most fatal form of the disease when untreated [1, 2]. The disease is caused by *Leishmania donovani* and *Leishmania infantum*, species which are characterized by parasite dissemination in organs such as the liver, spleen and bone marrow of the mammalian hosts [3, 4].

Since the treatment against VL is complex, including long therapeutic regimens, drug toxicity and high cost [5], a vaccine should be considered to prevent disease. It is known that patients who heal their infections develop a protective immunity against *Leishmania*, much in the same way as healthy subjects living in endemic areas of disease, and do not develop symptomatic VL [6]. The immune response associated with the protection is attributed to the development of a T helper (Th)1-type immunity, which is based on the production of cytokines, such as interferon-gamma (IFN-γ), tumor necrosis factor-alpha (TNF-α), interleukin (IL)-12, granulocyte-macrophage colony-stimulating factor (GM-CSF), among others, by CD4^+^ and CD8^+^ T cell subtypes. These molecules are capable of activating infected macrophages to produce nitric oxide (NO), which is responsible for intracellular amastigote death [7, 8]. By contrast, cytokines, such as IL-4, IL-10, IL-13, and transforming growth factor beta (TGF-β), inhibit the development of Th1 responses, allowing the occurrence of the active disease [9, 10].

In addition, peripheral blood mononuclear cells (PBMCs) collected from patients with subclinical or asymptomatic infection respond to stimulation with leishmanial antigens, producing IFN-γ and IL-12. However, those developing acute VL present cells did not respond to stimulation with *Leishmania* antigens, nor did they proliferate and produce cytokines like IFN-γ [6, 11, 12]. In this context, this patient class presents high levels of IL-10, with the presence of enhanced IL-10 messenger ribonucleic acid (mRNA) expression in infected organs [13].

A vaccine candidate against VL should be able to induce long-lasting protective immunity in the mammalian hosts by inducing both T cell subtypes, which could be boosted by natural infections, thus reducing the number of doses necessary to guarantee protection against infection. In addition, this candidate should be effective against different *Leishmania* species, cheap and easy to produce [14]. However, these requirements are difficult to obtain, since most of the molecules tested are composed by immunogens that offer species-specific protection and present a high cost of production and/or a need to associate immune adjuvants to induce protection [15–18]. Regarding the use of adjuvants, the regulation of these compounds for humans is far more rigorous than those applied to veterinary vaccines, making it difficult to obtain a highly immunogenic composition to be used in these mammalian hosts [19].

Phage display is a high-throughput technology able to identify novel molecules to be used in immunological applications, such as vaccine candidates [20, 21], diagnostic markers [22, 23] and/or immunotherapeutic targets against diseases [24–26]. Regarding vaccines, phage molecules themselves act as immune adjuvants in the immunized mammalian hosts, eliminating the need to incorporate this product in the formulation [27, 28]. For instance, a vaccination protocol using two phage clones selected by antibodies in VL dog sera was performed in murine models, and both immunogens were protective against *L. infantum* or *L. amazonensis* challenge infection [29, 30]. In these studies, no adjuvant was associated with the phages before their administration in BALB/c mice, and these phages were considered to be immunogenic carriers of the foreign peptides present in the viral capsid. This strategy is deemed to be responsible for guaranteeing a protective immune response against the infection caused by distinct *Leishmania* species.

In this context, in the current study phage display technology was used to screen specific peptides against immunoglobulin G (IgG) antibodies derived from asymptomatic and symptomatic VL patients, which were separated from IgG purified from non-infected subjects. After the biopanning cycles, target peptides, called mimotopes, were used to stimulate PBMCs from non-infected subjects living in endemic areas of disease and from VL patients, aiming to select those able to induce higher levels of IFN-γ and a lower production of IL-10, based on their calculated selectivity and specificity. Selected clones with the highest IFN-γ/IL-10 ratios were used to immunize BALB/c mice, which were later challenged with *L. infantum* promastigotes. This new selection strategy presented here can be considered useful to identify vaccine candidates against VL, as it represents the first proof-of-concept for the discovery of novel immunogens able to induce specific Th1 responses through the use of phage-displayed peptides.

## Methods

### Blood samples

Peripheral blood samples were collected with (heparin) or without anticoagulant from non-infected subjects (*n* = 10; 7 males and 3 females, ranging from 24 to 51 years in age) living in an endemic area of disease (Belo Horizonte, Minas Gerais, Brazil), and from VL patients. Non-infected subjects presented no clinical sign of disease at the moment of sample collection and showed negative serological results when the Kalazar Detect™ Test (InBios® International, Seattle, USA) was applied. Symptomatic VL patients (*n* = 10; 6 males and 4 females, ranging from 22 to 56 years in age) presented anemia, hepatomegaly and splenomegaly, and were diagnosed by polymerase chain reaction (PCR), targeting *L. infantum* kinetoplastid deoxyribonucleic acid (kDNA) in their spleen and/or bone marrow aspirates. Asymptomatic VL patients (*n* = 7; 5 males and 2 females, ranging from 27 to 49 years in age) showed positive serological and parasitological results when the Kalazar Detect™ Test and PCR technique, respectively, were applied; however, they presented no clinical signal of the disease.

### Biopanning cycles on phage display

To perform the phage display technology, a purification of IgG antibodies of VL patients and healthy subjects sera was performed by coupling them onto magnetic microspheres conjugated to protein G (Dynabeads, Invitrogen, Carlsbad, California, USA), as described elsewhere [31]. To carry out the biopanning cycles, 10^12^ viral particles of the bacteriophage library, containing randomly constrained peptides fused to the pIII coat protein of M13 filamentous phages (Ph.D.®-C7C library, New England BioLabs, Ipswich, Massachusetts, USA) were diluted in 250 μl of a solution consisting of 50 mM Tris-HCl pH 7.5, 150 mM NaCl, and 0.1% Tween 20 buffer (TBS-T). The mixture was incubated for 30 min, at room temperature, with microspheres coupled to the purified IgG antibodies from healthy subjects for separation, which were magnetically captured. The remaining phages in the supernatant were recovered and transferred to a new tube, at which time they were submitted to positive selection using IgG of VL patients. The supernatant was removed, and the bound phages were washed five times in 1 ml of TBS-T, at which time they were eluted in 500 μl 0.2 M glycine buffer, pH 2.0. Next, 75 μl 1 M Tris-base pH 9.0 were added to neutralize the acidic pH of the solution. After the selection, 76 phage clones were individually amplified in LB medium using sterile culture microplates (96-well microtest TM plate, BD Falcon TM clear, BD Biosciences, San Diego, California, USA). Sequencing was performed by capillary electrophoresis on the ABI 3130 equipment by BigDye v 3.1 and POP7 polymer (Myleus Biotechnology®, Belo Horizonte, Minas Gerais, Brazil). A sequence scanner software (Applied Biosystems, Foster City, California, USA) was employed to analyze the AB1 output files. Of the 76 clones, 17 presented valid peptide sequences and were selected to stimulate PBMCs.

### PBMCs culture and cytokine assay

PBMCs were purified from VL patients and healthy subjects by density centrifugation through Ficoll-Hypaque (GE Healthcare Bio-Sciences AB, Uppsala, Sweden). Cells (10^7^) were cultured in Roswell Park Memorial Institute (RPMI) 1640 medium, which was supplemented with 20% inactivated fetal bovine serum (FBS, Sigma-Aldrich, St. Louis, Missouri, USA), 2 mM L-glutamine, 200 U/ml penicillin, 100 μg/ml streptomycin, 50 μM 2-mercaptoethanol, 1 mM sodium pyruvate, and 1× non-essential amino acid. PBMCs were plated in 48-well flat-bottomed tissue culture plates (Costar, Cambridge, MA, USA) and incubated in medium (control) or separately stimulated with the individual clones (10^10^ phages each) in a 5% CO_2_ humidified atmosphere at 37 °C for 5 days. A wild-type (WT) clone and a Random non-specific phage, which expressed a foreign peptide (GERQYFWYLSKK) that presented a lower than 30% similarity to *Leishmania* peptide sequences, were used as controls (10^10^ phages each). After, supernatants were collected, and IFN-γ and IL-10 levels were measured by a capture enzyme-linked immunosorbent assay (ELISA), using commercial kits (Human IFN-γ and IL-10 ELISA Sets, BD Biosciences, San Diego, California, USA) according to manufacturer’s instructions. Results were interpolated from a standard curve using recombinant cytokines and expressed in pg/ml. After immunogenicity experiments, two clones, B1 and D11, were selected, based on their higher selectivity and specificity values, and were tested in the in vivo experiments.

### Bioinformatics assays

The constrained Ph.D. library used for peptide selection presents conformational peptides with cysteine residues in the borders; therefore, analyses were performed using the 7-mer peptide sequence and the amino acid sequence (AC-XXXXXXX-CGGGS) contained in the fusion with the pIII bacteriophage capsid protein, as described elsewhere [32]. Sequences were deduced based on the nucleotides through the Expasy server (http://web.expasy.org/translate/) and were analyzed by the Basic Local Alignment Search Tool (BLAST) program (http://blast.ncbi.nlm.nih.gov/Blast.cgi) against proteins and motifs of the GenBank database. The putative protein function was predicted by BLAST search alignment in the UNIPROT server (http://www.uniprot.org/). Physicochemical properties of B1 and D11 phage-exposed peptides, such as molecular weight, hydrophobicity, and net charge were predicted, using the Compute pI/Mw tool (http://web.expasy.org/compute.pi/) and the antimicrobial peptide database server (http://aps.unmc.edu/AP/prediction/prediction main.php).

### Parasite, immunization, and infection

*Leishmania infantum* (MHOM/BR/1970/BH46) was used. Parasites were cultured at 24 °C in complete Schneider’s medium (Sigma-Aldrich, St. Louis, Missouri, USA), which was added with 20% FBS, 20 mM L-glutamine, 200 U/ml penicillin, and 100 μg/ml streptomycin, pH 7.4. The soluble *Leishmania* antigenic extract (SLA) was prepared as described previously [33]. For the vaccination experiments, BALB/c mice (*n* = 16 per group) were inoculated subcutaneously in their left hind footpad with the WT, Random, B1, or D11 clones (10^10^ phages each) or received saline. Three doses were administered at 14-day intervals. 30 days after the last immunization, animals (*n* = 8 per group) were euthanized for the analysis of the immune response elicited by vaccination. At the same time, the remaining animals (*n* = 8 per group) were infected subcutaneously in their right hind footpad with 10^7^ *L. infantum* stationary-phase promastigotes and were followed up for 60 days.

### Parasite load by a limiting dilution assay and real-time polymerase chain reaction (RT-PCR)

To evaluate the protection induced by the immunogens, the liver, spleen, draining lymph nodes (dLNs) and bone marrow (BM) of the infected and immunized animals were collected, and the parasite burden was evaluated by a limiting dilution technique, as described elsewhere [34]. Briefly, the organs were weighed and homogenized using a glass tissue grinder in sterile phosphate buffered saline (PBS). Tissue debris were removed by centrifugation at 150× *g*, and cells were concentrated by centrifugation at 2000× *g*. Pellets were re-suspended in 1 ml of Schneider’s medium together with 20% FBS, of which 220 μl were plated onto 96-well flat-bottom microtiter plates (Nunc, Nunclon, Sigma-Aldrich, St. Louis, Missouri, USA) and diluted in log-fold serial dilutions, using supplemented Schneider’s medium, to a 10^−1^ to 10^−12^ dilution. Each sample was plated in triplicate and read 7 days after the beginning of the culture, at 24 °C. Results were expressed as the parasites’ percentage, which was normalized by the values obtained in the control (saline) group. The parasite load was also investigated in the animals´ spleen by the real time-polymerase chain reaction (RT-PCR) technique, as described elsewhere [31]. Results were expressed by the number of parasites per 1000 nucleated cells in the experimental groups.

### Cellular and humoral response

To evaluate the immunogenicity of the phage clones before and after infection, their spleen cells were plated in 24-well plates (Nunc), in duplicate. Cells (5 × 10^6^) were incubated in RPMI 1640 medium or separately stimulated with each clone used in the immunization (10^10^ phages each) or *L. infantum* SLA (25 μg/ml), for 48 h at 37 °C in 5% CO_2_. IFN-γ, IL-4, IL-10, IL-12, and GM-CSF production was measured in the cell supernatant by using commercial kits (Pharmingen®, BD Biosciences, San Diego, California, USA), according to manufacturer’s instructions. The nitrite production was investigated by the Griess reaction and results were expressed as micromolar. To evaluate the participation of CD4^+^ and CD8^+^ T cells in the IFN-γ production, spleen cells of the animals of the B1 and D11-immunized groups were in vitro stimulated and incubated in the presence of monoclonal antibodies (mAb) against mouse IL-12 (C017.8), CD4 (GK 1.5) or CD8 (53–6.7) (5.0 μg/ml, in all cases). Appropriate isotype-matched controls [rat IgG2a (R35–95) and rat IgG2b (95–1)] were used (Pharmingen®, BD Biosciences, San Diego, California, USA). The humoral response was investigated in the animals before and after infection. For this, sera samples were collected and phage and parasite-specific IgG1 and IgG2a antibody levels were measured by an ELISA technique, as described elsewhere [35].

### Flow cytometry

A flow cytometry assay was performed in the spleen cells of the infected and immunized animals, aiming to evaluate the IFN-γ, TNF-α and IL-10-producing CD4^+^ and CD8^+^ T cell frequency after stimulation using *L. infantum* SLA, as described elsewhere [36]. Results were expressed as indexes, which were calculated by dividing the cytokine-producing CD4^+^ and CD8^+^ T cell percentage in the stimulated cultures by the values obtained with unstimulated cultures (control).

### Cloning and purification of a hypothetical protein

Bioinformatic assays indicated a *Leishmania* hypothetical protein as expressing the B1 phage-exposed peptide. In this context, this molecule (XP_001470472.1) was cloned using the genomic *L. infantum* DNA by PCR technique. The recombinant protein was expressed in an *Escherichia coli* M15 strain by adding 1.0 mM isopropyl-β-D-thiogalactopyranoside (IPTG, Promega, Montreal, Canada) for 3 h at 37 °C. For purification, bacteria were lysed and the product was centrifuged at 13,000×*g* for 20 min at 4 °C. The recombinant protein (rLiHyp) was purified under non-denaturing conditions using a His-Trap column (GE Healthcare Life Science, Pittsburgh, USA), which was attached to a fast protein liquid chromatography (FPLC, GE Healthcare Life Science) system.

### ELISA experiments

The recombinant *Leishmania* hypothetical protein (rLiHyp) was used in ELISA assays (0.5 μg per well) in reaction to the B1 phage-vaccinated mice sera, as well as by those derived non-immunized (naive) mice, WT or Random-vaccinated animals (all 1:100 diluted in TBS-T). An anti-mouse IgG horseradish-peroxidase conjugated antibody (1:10,000 diluted in TBS-T) was used, and reactions were developed by incubation with a solution composed by H_2_O_2_, ortho*-*phenylenediamine and citrate-phosphate buffer, pH 5.0. The optical density (OD) values of the samples were read in an ELISA microplate spectrophotometer (Molecular Devices, Spectra Max Plus, Sunnyvale, California, USA) at 492 nm (nm).

### Immunoblotting assay

To evaluate the specificity of the anti-B1 phage antibody to *Leishmania* proteins, an immunoblotting experiment using rLiHyp and *L. infantum* SLA was performed. For this, the recombinant protein and soluble antigenic extract (10 and 20 μg, respectively) were submitted to a 12% sodium dodecyl sulfate polyacrylamide gel (SDS-PAGE) and blotted onto a nitrocellulose membrane (0.2 μm pore size, Sigma-Aldrich, St. Louis, Missouri, USA). Next, membranes were blocked with TBS-T plus 5% bovine serum albumin (BSA) for 1 h, and were independently incubated with pools of sera from non-immunized mice (*n* = 6) or B1 phage-vaccinated animals (*n* = 6), all 1:100 diluted in TBS-T for 2 h. As a secondary antibody, blots were incubated with peroxidase conjugated IgG anti-mouse (1:10,000; Sigma-Aldrich, St. Louis, Missouri, USA) for 2 h. Reactions were developed by adding chloronaphtol, diaminobenzidine, and H_2_O_2_, and were stopped by adding distilled water.

### Statistical analysis

The results were entered into Microsoft Excel (version 10.0) spreadsheets and analyzed by GraphPad Prism™ (version 6.0 for Windows). Statistical analyses were performed by one-way analysis of variance (ANOVA), followed by the Bonferroni’s *post-hoc* test for comparisons between the groups. Differences were considered significant with *P* < 0.05. The vaccination experiments were repeated and the results were similar between them. Data showed in this study are representative of the first experiment. Results obtained in the second study are shown in Additional file 1: Figure S1.

## Results

### Selecting target clones by phage display and validating them by means of immunological assays

In the present study, an experimental strategy using antibodies purified from the sera of non-infected subjects, as well as from asymptomatic and symptomatic VL patients, was performed, aiming to select new candidates to be tested as immunogens against disease. After three biopanning cycles, 76 clones were selected and sequenced, and 17 valid peptide sequences were deduced by the Expasy server. An alignment showed no consensus motif among sequences, and none of these were non-specific binders to the commons reagents used in the selection cycles (data not shown). The clones were then used to stimulate PBMCs from both non-infected subjects and VL patients, and IFN-γ and IL-10 levels were measured in the cellular supernatants. As controls, WT and Random phages, as well as *L. infantum* SLA, were used to stimulate cells.

**Fig. 1:**
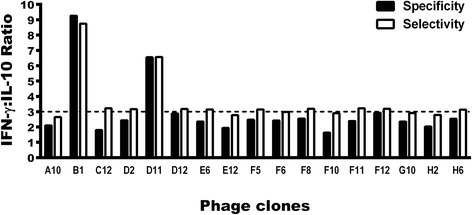
Evaluation of selectivity and specificity of the phage clones. PBMCs were purified from blood samples from asymptomatic and symptomatic VL patients and non-infected subjects. Cells were non-stimulated (medium) or stimulated with each clone, as well as by Wild-type (WT) and Random clones (10^10^ phages each), for 48 h at 37 °C in 5% CO_2_. IFN-γ and IL-10 levels were measured in the culture supernatants by an ELISA capture. Black bars indicate the specificity of the clones, which was calculated by dividing the IFN-γ and IL-10 values obtained of each evaluated clone through respective values of these cytokines obtained after the WT phage stimulus, using PBMCs from healthy subjects. With the corrected values, the ratio between the IFN-γ and IL-10 levels with these new results was calculated, and the specificity of the each clone was defined and is shown here. White bars indicate the selectivity, which was calculated by dividing the IFN-γ and IL-10 values obtained of each evaluated clone through respective values of these cytokines obtained after the Random phage stimulus, using PBMCs from VL patients. With the corrected values, a ratio between the IFN-γ and IL-10 levels with these new results was calculated, and the selectivity of each clone was defined and is shown here

In an attempt to select the best immunogenic molecules, the specificity and selectivity were calculated for each one of the 17 clones, as compared to results obtained using the WT and Random phages as stimuli. An experimental strategy similar to this, developed by Costa et al. [23], was employed. For this, specificity was presented as the ability of each clone to bind to its target based on the presence of phage surface-displayed peptide. Values were calculated by determining the ratio between IFN-γ and IL-10 levels, which were obtained after the stimulation of PBMCs from non-infected subjects through the respective values obtained using the WT phage stimulus. A new ratio was then calculated and results were defined as the specificity of each clone. The selectivity was presented as the ability of each clone to bind to its target based on the mixture of different molecules. Values were calculated by determining the ratio between the IFN-γ and IL-10 levels, which were obtained after stimulation of PBMCs from VL patients through the respective values obtained using the Random phage stimulus. A new ratio was then calculated and results were defined as the selectivity of each clone. As a result, B1 and D11 clones presented ratios between IFN-γ and IL-10 cytokines of higher than 3.0 and were selected to be tested in the in vivo experiments (Fig. 1).

**Fig. 2:**
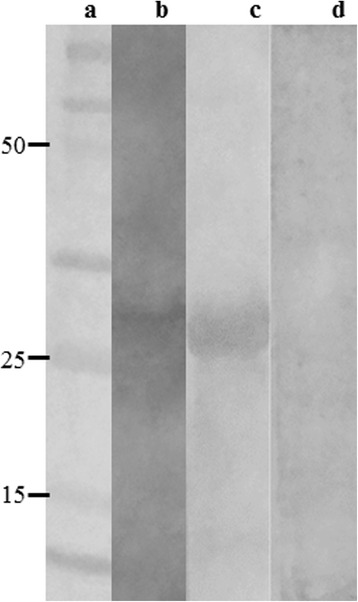
Immunoblotting assays. A molecular weight marker (Lane a), 20 μg *L. infantum* SLA (Lane b) and 10 μg rLiHyp (Lanes c, d) were electrophoresed on a 20% SDS-PAGE and blotted onto nitrocellulose membranes. Blots were incubated with pools of sera from B1 phage-vaccinated mice (Lanes b and c) or from naive mice (lane d), and were revealed by adding chloronaphtol, diaminobenzidine, and H_2_O_2_. A scan from the blots is shown here. The black arrow indicates the rLiHyp protein (~28.0 kDa)

Bioinformatics assays were used to evaluate the physicochemical properties of B1 and D11 clones, when net charge, molecular weight, and hydrophobicity were obtained: +1 and 0; 1396.6 and 1254.5; and 42 and 50%, respectively. Using BLAST analyses to predict target peptides in *Leishmania* proteins, the B1 phage-exposed peptide proved to be present in a hypothetical protein (XP_001470472.1) and in the putative inositol polyphosphate kinase-like protein (XP_001464217.1), while the D11 phage-exposed peptide was identified in both the putative kinase protein (XP_001470438.1) and the permease protein (XP_003392714.1). To partially validate our findings through the application of bioinformatics, the hypothetical protein amino acid sequence (XP_001470472.1), which was identified as expressing the B01 mimotope, was cloned and its recombinant version (rLiHyp) was employed in ELISA and immunoblotting assays, in an attempt to verify the antigenicity of anti-B1 clone antibodies. Regarding ELISA experiments, high reactivity was found in the sera of B1 phage-immunized animals when the recombinant protein was used as an antigen in the plates. In this context, the mean ± standard deviation of the OD values for the sera of these animals was of 0.550 ± 0.044, while the use of sera from naive, WT or Random phage-vaccinated mice presented the results of 0.022 ± 0.04, 0.040 ± 0.008 and 0.035 ± 0.012, respectively. An immunoblotting assay also showed that B1 phage-immunized animal sera reacted with *L. infantum* SLA and rLiHyp protein (Fig. 2), showing the specificity of the anti-B1 phage antibodies in recognizing this *Leishmania* protein.

### Immunization with B1 and D11 clones protect mice against *L. infantum* infection

**Fig. 3:**
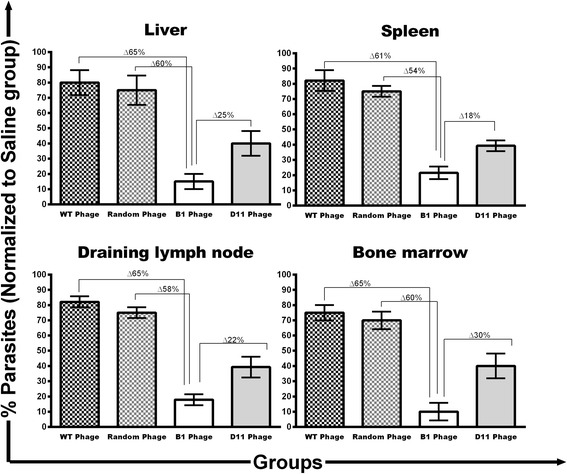
Parasite burden in infected and vaccinated animals evaluated by a limiting dilution technique. BALB/c mice were immunized with the Wild-type (WT), Random, B1, or D11 phage clones and later infected with 10^7^ *L. infantum* stationary promastigotes*.* The parasite loads from each group were normalized with the values found in the saline group. Results of the parasites’ percentage in the liver, spleen, draining lymph nodes and bone marrow, as well as the differences between groups are shown. Bars represent the mean ± standard deviation. Data shown in this study are representative of two independent experiments, which presented similar results

**Fig. 4:**
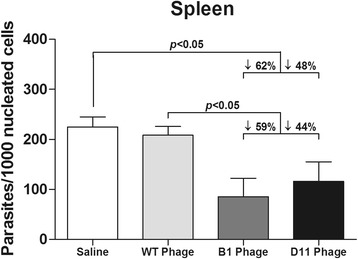
Splenic parasite burden evaluated by RT-PCR. Mice received saline or were immunized with the Wild-type (WT), B1 or D11 clones, and were later infected with 10^7^ *L. infantum* stationary promastigotes. The splenic parasite load was then evaluated by a RT-PCR technique. Results showing the parasite load, which was based on the number of parasites per 1000 nucleated cells, are shown here, as are the differences in the parasites’ percentage between the groups. Bars represent the mean ± standard deviation

The B1 and D11 clones were administered in BALB/c mice, which were later challenged with *L. infantum* promastigotes. Sixty days after infection, the organs of the animals were collected and the parasite load was evaluated by a limiting dilution technique. Vaccinated mice with B1 or D11 clones, when compared to the WT group, showed a significant reduction in the parasite percentage in the liver (65% and 40% reductions, respectively) (*F*_(4,15)_ = 16.68, *P* < 0.0001), spleen (61 and 43%, respectively) (*F*_(4,15)_ = 33.36, *P* < 0.0001), draining lymph nodes (dLN, 65 and 43%, respectively) (*F*_(4,15)_ = 48.26, *P* < 0.0001) and bone marrow (BM, 65 and 35%, respectively) (*F*_(4,15)_ = 19.44, *P* < 0.0001) (Fig. 3). When compared to the data from the Random phage group, B1 or D11 phages-immunized animals showed a significant reduction in parasite percentage in the liver (60 and 35%, respectively) (*F*_(4,15)_ = 16.68, *P* < 0.0001), spleen (54 and 36%, respectively) (*F*_(4,15)_ = 33.36, *P* < 0.0001), dLN (58 and 36%, respectively) (*F*_(4,15)_ = 48.26, *P* < 0.0001) and BM (60 and 30%, respectively) (*F*_(4,15)_ = 19.44, *P* < 0.0001). Data of parasite burden obtained in the second experiment are also shown (Additional file 1: Figure S1). Overall, B1 clone-immunized mice showed better protection in relation to the D11 phage. These data were corroborated by a RT-PCR assay, since immunization with B1 and D11 clones induced parasite percentage reductions in the spleen of the animals, as compared to data from saline, WT and Random groups (Fig. 4).

### B1 and D11 phages-immunized mice develop a Th1 response before infection, which is maintained after challenge

**Fig. 5:**
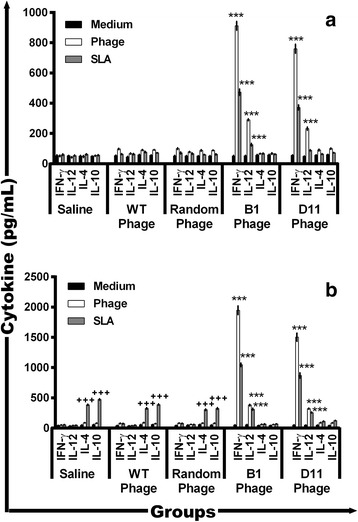
Cellular response before and after *L. infantum* infection. Splenocytes were obtained from the spleen of mice, 30 days after last immunization and 60 days after infection. Cells were stimulated with medium or stimulated with *L. infantum* SLA (25 μg/ml), or with each clone (10^10^ phages) used in the immunization (group saline was stimulated with a phages mix), for 48 h at 37 °C in 5% CO_2_. IFN-γ, IL-12, GM-CSF, IL-4, and IL-10 levels were measured in culture supernatants by ELISA before (**a**) and after (**b**) infection. Bars represent the mean ± standard deviation. ^***^ *P* < 0.0001 (a statistically significant difference in relation to the saline, WT and Random groups). ^+++^ *P* < 0.0001 (a statistically significant difference in relation to the B1 and D11 groups)

**Fig. 6:**
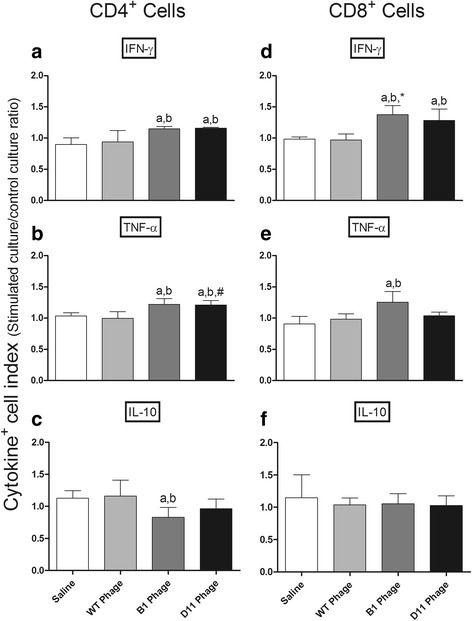
Intracytoplasmic cytokine-producing T cell frequency after stimulus with *L. infantum* SLA. Cytokine indexes were calculated as the ratio of cytokine^+^ cells observed in SLA-stimulated cultures divided by the control culture (SLA/CC ratio). Mice received saline (Saline: white rectangle), or were immunized with wild-type (WT Phage = light grey bars), B1 (B1 Phage = dark grey bars) or D11 phages (D11 Phage = black bars). Later, they were challenged with *L. infantum* promastigotes and, 60 days after infection, their splenocytes were collected and in vitro stimulated with *L. infantum* SLA (25 μg/ml). Results were reported as cytokine indexes (Stimulated culture/Control culture ratio) for IFN-γ, TNF-α, and IL-10 in **a**, **b** and **c**, respectively, for CD4^+^ T cells and in **d**, **e** and **f**, respectively, for CD8^+^ T cells, and were expressed as mean plus standard deviation of the groups. Letters above bars indicate a statistically significant difference in relation to the saline and WT phage groups, respectively (*P* < 0.05); asterisk indicates a statistically significant difference between CD8^+^ and CD4^+^ IFN-γ^+^ producing T cells (*P < 0.05); # indicates a statistically significant difference between CD4^+^ and CD8^+^ TNF-α^+^ producing T cells (^#^ *P* < 0.05)

The immunogenicity of B1 and D11 clones was evaluated in vaccinated mice 30 days after the last vaccine dose and before infection, as well as 60 days after challenge (Fig. 5). After in vitro stimulation with phages or *L. infantum* SLA, spleen cells from the animals of the B1 and D11 groups produced significantly higher levels of IFN-γ (*F*_(4,15)_ = 489.3, *P* < 0.0001 and *F*_(4,15)_ = 248.3, *P* < 0.0001, respectively) and IL-12 (*F*_(4,15)_ = 382.3, *P* < 0.0001 and *F*_(4, 15)_ = 31.24, *P* < 0.0001, respectively) than those from control mice. Low and insignificant levels of IL-4 (*F*_(4,15)_ = 17.48, *P* = 0.0211; *F*_(4,15)_ = 3.988, *P* = 0.0213) and IL-10 (*F*_(4,15)_ = 31.06, *P* = 0.0221; *F*_(4,15)_ = 6.990, *P* = 0.0222) were observed in all groups. After challenge, the Th1 immune profile was maintained in the B1 and D11 phage-vaccinated animals. However, mice from the control groups presented a higher production of IL-4 and IL-10 towards a Th2 response profile (Fig. 5). Data of cellular response, which were obtained before and after infection in the second experiment, are also shown (Additional file 1: Figure S1). In addition, the intracytoplasmic cytokine response, which was evaluated by a flow cytometry, also showed that both B1 and D11-vaccinated mice presented higher levels of anti-leishmanial IFN-γ^+^ and TNF-α^+^-producing CD4^+^ and CD8^+^ T cells, which were associated with a low presence of IL-10^+^ T cells, when compared to results obtained in the saline, WT and Random groups (Fig. 6).

**Fig. 7:**
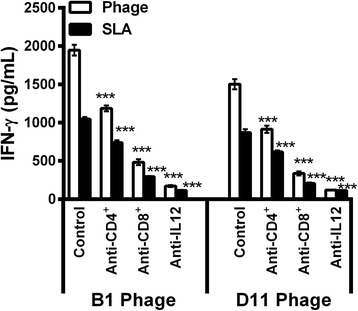
Involvement of CD4^+^ or CD8^+^ T cells in IFN-γ production. Spleen cells of mice that were immunized with B1 or D11 clones, and later infected, were in vitro stimulated with the respective clones (1 × 10^10^ phages) or with SLA (25 μg/ml) for 48 h at 37 °C in 5% CO_2_. After, these were maintained in the absence (control) or incubated in the presence of monoclonal antibodies against mouse IL-12, CD4^+^ or CD8^+^ (5 μg/ml each). The IFN-γ production in the supernatants was evaluated, and the results are shown here. Bars represent the mean ± standard deviation. ***P < 0.0001 (a statistically significant difference in relation to the control group)

**Fig. 8:**
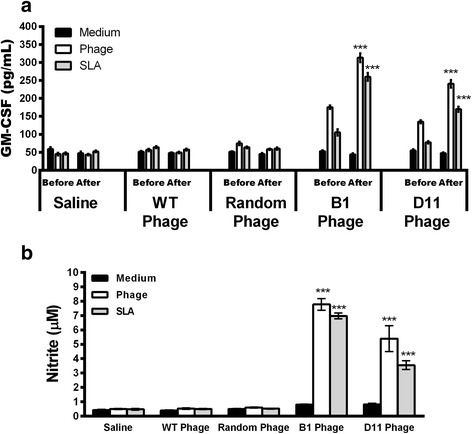
Macrophage activation in the infected and vaccinated animals. Spleen cells from mice that received saline or were immunized with wild-type (WT), Random, B1 or D11 phage clones, and later infected with *L. infantum* promastigotes, were in vitro stimulated with SLA (25 μg/ml) or with the respective clone (1 × 10^10^ phages) for 48 h at 37 °C in 5% CO_2_. The GM-CSF production (**a**) was evaluated in the vaccinated (Before) as well as in the infected (After) animals, whereas the nitrite secretion (**b**) was evaluated after infection. Bars represent the mean ± standard deviation. ***P < 0.0001 (a statistically significant difference in relation to the saline, WT and Random groups)

**Fig. 9:**
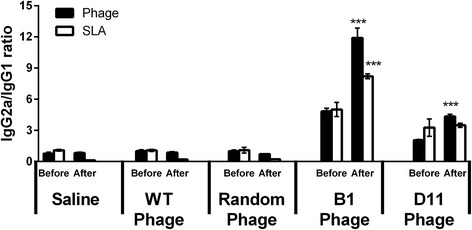
Phage and parasite-specific humoral response before and after infection. BALB/c mice received saline or were immunized with Wild-type (WT), Random, B1 or D11 clones. Thirty days after the last immunization, sera samples were collected (Before). In addition, remaining animals were challenged with *L. infantum* promastigotes and followed up for 60 days, at which time sera samples were collected (After). The anti-phage and anti-parasite IgG2a and IgG1 isotype antibody levels were obtained, and the ratios between IgG2a and IgG1 results were calculated and are shown here. Bars represent the mean ± standard deviation. ***P < 0.0001 (a statistically significant difference in relation to the saline, WT and Random groups)

The involvement of CD4^+^ and CD8^+^ T cells in the phage and parasite-specific IFN-γ production in B1 and D11 phage-vaccinated mice was also evaluated (Fig. 7). IFN-γ levels were significantly decreased when both anti-CD4 (*F*_(2,9)_ = 198.0, *P* < 0.0001) and anti-CD8 (*F*_(2,9)_ = 139.8, *P* < 0.0001) monoclonal antibodies were used in the spleen cell cultures. It is important to note that IFN-γ production was higher after the application of the anti-CD4 antibody than after that of the anti-CD8 antibody in the vaccinated groups. The presence of IFN-γ stimulates the production of NO and other molecules by phagocytic cells, thereby assisting in parasite control. In addition, GM-CSF, a cytokine related to macrophage activation and resistance in murine models against different *Leishmania* spp., is also required to protect against infection. To evaluate the macrophage activation in the infected and phage-vaccinated animals, GM-CSF and nitrite levels were measured in cell supernatants. In the results, significantly higher levels of GM-CSF (*F*_(4,15)_ = 280.5, *P* < 0.0001; *F*_(4,15)_ = 207.9, *P* < 0.0001) (Fig. 8a) and nitrite (*F*_(4,15)_ = 59.58, *P* < 0.0001; *F*_(4,15)_ = 296.7, *P* < 0.0001) (Fig. 8b) were observed in B1 and D11 phage-immunized mice, when compared to control groups, thus showing the importance of these molecules in the control of the parasitism against *Leishmania*. The B1 and D11 phages-vaccinated mice exhibited a humoral response with a predominance of phage- and SLA-specific IgG2a isotype, when compared to the IgG1 levels found before and after infection. Results shown as ratios between IgG2a and IgG1 levels demonstrate that mice from the B1 and D11-vaccinated groups presented a higher IgG2a/IgG1 ratio, whereas animals from the saline, WT and Random groups showed a mixed IgG2a/IgG1 profile, with values near of 1.0 (Fig. 9). Data of humoral response, which were obtained before and after infection in the 2nd experiment, are also shown (Additional file 1: Figure S1).

## Discussion

In recent decades, several antigens, such as A2 [37], nucleoside hydrolase (NH)36 [38], ribosomal proteins [35, 39] and cyclophilin [40] among others, have been considered candidates to produce a protective vaccine against leishmaniasis. Despite the large number of molecules tested and vaccination protocols employed, no effective vaccine against human disease has been achieved. As a consequence, new candidates based on phage-exposed peptides have been successfully identified by phage display, thus resulting in new diagnostic markers, vaccine candidates, and/or immunotherapeutic targets against diseases [41–44]. In this light, our research group has successfully applied this technology to select new candidates for serodiagnosis [23, 45] and as vaccine candidates [46, 47] against canine VL.

The development of a human vaccine against leishmaniasis is possible, since approximately 80% of *L. donovani*-infected patients clear their parasites spontaneously before developing the active disease, and they present protective immunity against reinfection [46]. In addition, nearly 85% of all treated and cured VL patients showed resistance against *L. donovani* reinfection [47, 48]. The current study has proposed a new strategy to identify phage-displayed peptides as immunogens to be tested as vaccine candidates against VL by mimicking the immune response of asymptomatic patients and subjects living in endemic areas of the disease. Here, new targets were selected by the cytokine profile (i.e. a polarized Th1 response, as evidenced by higher IFN-γ/IL-10 ratio) induced in human PBMCs from asymptomatic VL patients and non-infected individuals. These were successfully tested in an in vivo murine model against experimental infection by the parasite.

The selection strategy used to identify new immunogenic targets based on phage molecules can be considered valid, since these products are not infective or pathogenic to mammalian hosts, but they do replicate inside the host cells, improving the quality and intensity of the generated immune response [49]. In addition, phage-exposed peptides are robust, stable to harsh conditions and highly immunogenic, and present five copies in each viral particle, in a population of millions of phages, but without interfering in the infectivity of these agents [50–53]. Moreover, the production of the compounds is considered easier and cheaper, when compared to the synthesis of peptides or production of recombinant proteins [54].

Adjuvants are defined as molecules able to activate the innate immunity receptors expressed in the antigen-presenting cells of the mammalian hosts, which take up and present the antigens to T helper cells, improving the efficacy of the antigen-specific response [55]. Most studies evaluating vaccine candidates against leishmaniasis used recombinant molecules associated with Th1-type adjuvants [56–60]. However, the use of such products presents disadvantages, mainly due to the low number of compounds available and licensed for human use, in addition to the higher cost of production of the vaccine formulation. However, in the current study, no adjuvant was added to the B1 and D11 clones, since the phage molecule is able to stimulate through the development of a Th1 response primed by the production of IFN-γ and IL-12 due to the presence of stimulatory non-methylated cytosine-phosphate-guanosine (CpG) motifs in their viral genome, which activate the immune system of the mammalian hosts through Toll-like receptors [52, 61–64].

We have successfully shown the protective efficacy of B1 and D11 clones against *L. infantum* infection in the immunized mice, compared to control groups, including WT phage and a Random phage-fused mimotope. Since phages displaying peptides can induce humoral and cell-mediated immune responses without the need for an adjuvant, results obtained here show the development of a specific Th1 response in the B1 and D11 phage-vaccinated animals, which was maintained after infection. Indeed, spleen cells of vaccinated mice mounted an in vitro specific production of IFN-γ, IL-12 and GM-CSF, which was combined with low levels of IL-4 and IL-10, in addition to the predominance of parasite-specific IgG2a antibodies. This immunogenic effect was reflected by protection against *L. infantum* infection, since significant reductions in the parasite burden in different organs was reached, when compared to the control groups, thus showing the efficacy of these immunogens in protecting against challenge infection.

Understanding the factors mediating protective immunity against leishmaniasis, and whether the immune response can be mediated by memory T cells, will contribute to a long-term protection against parasite infection by means of the generation of long-lasting protective vaccines, since naturally acquired resistance to reinfection by *Leishmania* coincides with an ongoing primary infection. Studies have shown that T cells presenting functional attributes of memory cells are maintained in the absence or low presence of parasites, and this fact could represent long-lasting protection against reinfection [65]. In the present study, immunological memory induced by immunization using the B1 and D11 phages was not evaluated in the vaccinated animals. However, this procedure will be conducted in future works in an attempt to investigate the protective potential of these immunogens in animals against a more long-lasting infection. These aspects will certainly contribute to an improved design for vaccine candidates with respect to their ability to raise memory response, aiming to improve their protective performance against *L. infantum* infection.

## Conclusions

The present study’s data pointed out the immunological applications of B1 and D11 clones as vaccine candidates against VL. The employment of products able to offer protection against disease added support to further investigation, thus employing both phage clones and focusing on the perspectives of the rational improvement of the vaccine formulation, which might have a positive impact upon the management of this important but relevant and neglected disease.

## Additional file

Additional file 1: Figure S1. Parasitological and immunological evaluations obtained in the infected and/or vaccinated animals in the second vaccine experiment. BALB/c mice (*n* = 16, per group) were vaccinated subcutaneously in their left hind footpad with Wild-type (WT), Random, B1 or D11 phage clones. Additional mice received only saline (*n* = 16). Three doses were administered at 14-day intervals. 30 days after the last vaccine, animals (*n* = 8, per group) were infected in the right hind footpad with 1 × 10^7^ stationary promastigotes of *L. infantum*. Before and 60 days after challenge, the anti-phage and anti-parasite IgG2a and IgG1 isotype antibody levels were obtained, and the ratios between IgG2a and IgG1 results were calculated and are shown before (**a**) and after (**b**) infection. In addition, spleen cells were collected to evaluate the cytokine response, when they were incubated in complete RPMI 1640 medium (negative control) or in vitro stimulated with SLA (25 μg/ml) or with the respective clone (1 × 10^10^ phages), for 48 h at 37 °C in 5% CO_2_. IFN-γ, IL-12, GM-CSF, IL-4 and IL-10 levels were then measured by ELISA in the culture supernatants before (**c**) or after (**d**) infection. 6 days after challenge, the parasite burden was determined in the liver, spleen, draining lymph nodes and bone marrow of the animals, by a limiting dilution assay (**e**). Using the cell supernatants employed to evaluate cytokines, the nitrite production was also evaluated in this time (**f**). ^+^indicates a statistically significant difference in relation to the B1 and D11 phages groups (*P* < 0.0001). ^***^indicates a statistically significant difference in relation to the saline, WT and Random groups (*P* < 0.0001). (TIFF 139 kb)

## Abbreviations

BLAST: Basic Local Alignment Search Tool
BM: Bone marrow
DLNs: Draining lymph nodes
ELISA: Enzyme-linked immunosorbent assay
FBS: Fetal bovine serum
FPLC: Fast protein liquid chromatography
GM-CSF: Granulocyte-macrophage colony-stimulating factor
IFN-γ: Interferon-gamma
IgG: Immunoglobulin G
IL: Interleukin
IPTG: Isopropyl-β-D-thiogalactopyranoside
KDNA: Kinetoplastid deoxyribonucleic acid
MRNA: Messenger ribonucleic acid
NH: Nucleoside hydrolase
Nm: Nanometers
NO: Nitric oxide
OD: Optical density
PBMCs: Peripheral blood mononuclear cells
RLiHyp: Recombinant *Leishmania* hypothetical protein
RT-PCR: Real-time polymerase chain reaction
SDS-PAGE: Sodium dodecyl sulfate polyacrylamide gel
SLA: Soluble *Leishmania* antigenic extract
TGF-β: transforming growth factor beta
Th: T helper
TNF-α: tumor necrosis factor-alpha
VL: Visceral leishmaniasis
WT: Wild-type
